# Incidence of venous thromboembolism in northern Sweden (VEINS): a population-based study

**DOI:** 10.1186/1477-9560-12-6

**Published:** 2014-03-04

**Authors:** Magdalena Johansson, Lars Johansson, Marcus Lind

**Affiliations:** 1 Department of Public Health and Clinical Medicine, Skellefteå Research Unit, Umeå University, Skellefteå County Hospital, 931 86 Skellefteå, Sweden

**Keywords:** Venous thromboembolism, Venous thrombosis, Pulmonary embolism, Deep vein thrombosis, Risk factors, Incidence, Epidemiology, Adults

## Abstract

**Background:**

The reported incidence of venous thromboembolism (VTE) varies considerably among studies. The primary aim of this study was to describe the incidence of VTE in relation to age and sex. The secondary aim was to describe the risk factor pattern at the time of diagnosis.

**Methods:**

This retrospective, population-based cohort study included all adult residents in the County of Västerbotten in northern Sweden during the year 2006 (n = 204,836). All potential VTE events were manually validated and classified according to location. The presence of risk factors for VTE at the time of diagnosis was recorded.

**Results:**

We identified 517 adult individuals with potential VTE. Among these, 343 individuals (158 men and 185 women) had a verified VTE event in 2006. The mean incidence was 167 individuals per 100,000 person years; 155 for men and 180 for women. The mean age at diagnosis was 67.6 years in men and 72.5 years in women. The incidence of VTE increased with age. The incidence was highest in women aged 85 years or more. Pulmonary embolism with or without concurrent deep vein thrombosis was diagnosed in 161 individuals (46.9%); lower extremity deep vein thrombosis without concurrent pulmonary embolism was diagnosed in 157 individuals (45.8%); and VTE in another location was diagnosed in 25 individuals (7.3%). The most common risk factors for VTE were recent hospitalization and concurrent malignancy.

**Conclusion:**

The incidence of VTE was 167 per 100,000 person years and increased with age. The incidence was highest among older women. Pulmonary embolism was the most common form of VTE; it affected 47% of individuals with VTE. Malignancy and hospitalization were the most prevalent risk factors for VTE.

## Background

Lower extremity deep vein thrombosis (DVT) and pulmonary embolism are the two most common disease manifestations of venous thromboembolism (VTE). VTEs also manifest as thrombosis of the veins of the central nervous system, upper extremity DVT, and thrombosis of the veins of the abdomen, such as portal and renal vein thrombosis. Examples of previously described risk factors for VTE are hospitalization, immobilization, recent surgery, and malignancy [1]. The 30-day mortality was reported to be 6.4% for all patients with VTE, 9.7% for those with pulmonary embolism, and 4.6% for those with DVT [2].

The incidence of first-time VTE is reported to vary considerably; it ranges between 62 and 143 per 100,000 individuals per year [2-8]. Some reports describe variations in incidence among different ethnicities [9,10]. In previous studies, the incidence of first-time pulmonary embolism ranged between 19 and 50 per 100,000 per year. The incidence of first-time DVT ranged between 38 and 95 per 100,000 per year [2-8].

In Sweden, new diagnostic procedures were widely introduced at the end of the 1990s. Currently, VTE is routinely evaluated with computed tomography (CT), D-dimers, and clinical risk assessments, such as Well’s score. VTE prophylaxis is also widely used [11,12]. On the other hand, the autopsy rate has declined [13]. These changes may have had an impact on the VTE incidence.

The primary aim of this study was to describe the incidence of VTE in relation to age and sex. The secondary aim was to describe the risk factor pattern at the time of diagnosis.

## Methods

### Case identification and setting

Case identification was performed in the County of Västerbotten, a sparsely populated area located in northern Sweden. In 2006, the adult population (age 18 and over) was 204,836, and 22.6% was aged 65 years or more [14]. The vast majority of residents were Caucasian. All health care was administered by two district general hospitals, one university hospital, and their associated primary healthcare centers.

To include all potential VTE events, we performed an extensive search for VTE cases in the National Patient Registry that included ICD-10 diagnosis codes I26, I27.8, I27.9, I67.6, I80, I81, I82, O08.2, O08.7, O22.5, O22.8, O22.9 and O22.2, for inpatients, outpatients, and emergency department visits in all three hospitals in the County of Västerbotten for the year 2006. For comparison, we also performed a limited search that included only the ICD-10 diagnosis codes I26 and I80.1-9. We also searched the mortality registry maintained by the Swedish National Board of Health and Welfare to find individuals whose death certificates stated that the cause of death was a VTE event. To find additional cases of VTE that were either not coded or miscoded, we searched the anticoagulant registry, the radiology registry, and the diagnosis registry (ICD-10 codes I74.3 and K55.0) at one of the three hospitals in the area.

The study was approved by the Regional Ethics Review Board of Umeå University.

### Case validation and classification

All cases identified as potential VTEs were validated by reviewing the relevant medical records. Validation and classification of the VTE events were performed by three physicians (M.J., L.J., and M.L.). Ambiguities were resolved by discussion.

A pulmonary embolism was considered verified when confirmed by pulmonary angiography, CT, magnetic resonance imaging (MRI), high probability ventilation-perfusion, or at autopsy. An individual with a verified DVT and concomitant symptoms of pulmonary embolism was considered to have pulmonary embolism. A DVT of the lower or upper extremity was considered verified when confirmed by venography, ultrasonography, CT, MRI, or at autopsy. An abdominal VTE event was classified as verified when confirmed by venography, ultrasonography, CT, MRI, or at autopsy. A VTE of the central nervous system was considered verified when confirmed by CT, MRI, or at autopsy. In patients with previous VTEs, a recurrence of VTE required objective evidence, on an appropriate imaging scan, of a new thrombosis that was not identified during a previous imaging scan.

Individuals were classified into three groups according to the location of the VTE: a pulmonary embolism, a lower extremity DVT, or a VTE in another location. VTEs in other locations included an upper extremity DVT (defined as thrombosis of the deep upper extremity veins, the superior vena cava, the internal jugular vein, the subclavian vein, the innominate vein, or the axillary veins), a thrombosis of the veins of the abdomen, or a thrombosis of the veins of the central nervous system.

For each individual, we considered only the first verified VTE event during the study period. Individuals with multiple concurrent thrombosis locations were classified hierarchically into one of the following groups: 1: pulmonary embolism, 2: lower extremity DVT, or 3: VTE in another location. Individuals with a verified DVT and symptoms of pulmonary embolism were classified into the pulmonary embolism group. The VTE event was also classified as either a first-time VTE or a recurrent VTE. The event was classified as recurrent when at least one previous VTE event (before 2006) was documented.

A lower extremity DVT was classified as iliac when it was located at or above the inguinal ligament; it was classified as femoropopliteal when it was located in or above the popliteal vein, but distal to the inguinal ligament; and it was classified as a calf DVT when it was located below the popliteal vein. It was classified as muscular vein thrombosis when it was located in a muscular or perforating vein.

### Case characterization

The cases were characterized according to presence of risk factors for VTE at the time of diagnosis. We recorded the use of central venous lines, pharmacological VTE prophylaxis, or systemic hormone therapy (including contraceptives). We also extracted from the medical records data on recent hospitalization, recent surgery, recent immobilization for more than 48 hours, recent plaster cast treatment, pregnancy or postpartum (defined as the period within 60 days of delivery), recent trauma, and whether the patient had recently travelled for more than eight hours. Recent events were defined as those that occurred within 60 days prior to the onset of VTE symptoms. We also recorded the presence of a previous VTE, a family history of VTE in a first degree relative, and the presence and, when applicable, the type of coagulation disorder.

To assess the presence of malignant disease, we conducted a search of the Swedish Cancer Registry. Data for all malignant diseases detected in Swedish residents from 1958 to present were available in the registry. In Sweden, reporting to the registry is mandatory for health care providers; therefore, the coverage rate was high, estimated at 96% in 1998 [15].

### Statistical analysis

Patient characteristics are described as the mean and standard deviation or the number and proportion. The annual incidence of VTE was calculated for each sex and age strata by dividing the number of cases by the total number of inhabitants on the first of November, 2006.

## Results

### Incidence

**Figure 1:**
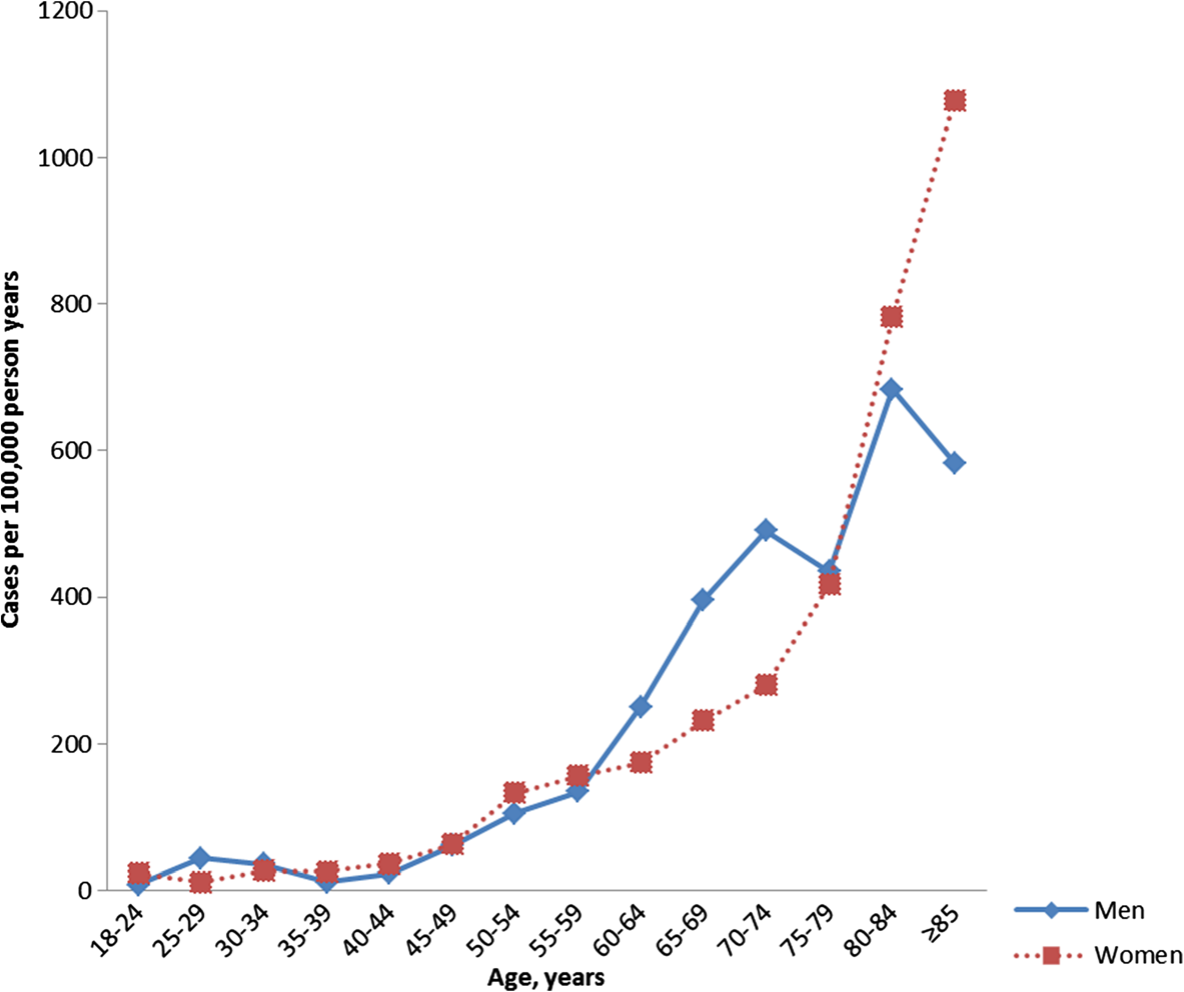
**Incidence of venous thromboembolism increases with age.** Men (blue); women (red).

We identified 343 adult residents of the County of Västerbotten with a verified diagnosis of VTE in 2006. The mean age was 67.6 years in men and 72.5 years in women. The overall proportions of women were 54% for those under age 80 years and 70% for those 80 and over. Of the patients with VTE, 281 (81.9%) had a first-time VTE event. Patient characteristics and risk factors at diagnosis for these 281 patients are shown in Table 1. The total incidence of VTE (per 100,000 per year) was 167 overall, 155 in men, and 180 in women. The incidence of VTE increased with age in both men and women (Figure 1). The incidence of pulmonary embolism was 78.6 per 100,000 per year. The incidence of lower extremity DVT without concurrent pulmonary embolism was 76.6 per 100,000 per year. The yearly incidence rates of upper extremity DVT, thrombosis of the veins of the central nervous system, and thrombosis of the veins of the abdomen were 4.9, 3.9, and 3.4 per 100,000, respectively. The incidence of first-time VTE was 137 per 100,000 per year.

**Table 1:** Patient characteristics and risk factors in 281 patients with first-time VTE, grouped by age

	**All (n = 281)**	**18-64 years (n = 92)**	**65-79 years (n = 100)**	**80 years and over (n = 89)**
Male	128 (45.6)	47 (51.1)	54 (54.0)	27 (30.3)
Mean age (years)	70.0 ± 15.2	52.3 ± 10.8	72.7 ± 4.2	85.5 ± 4.1
**Risk factors**				
Recent hospitalization^a^	120 (42.7)	39 (42.4)	44 (44.0)	37 (41.6)
Recent surgical intervention^a^	42 (14.9)	23 (25.0)	11 (11.0)	8 (9.0)
Recent immobilization >48 hours^a^	42 (14.9)	17 (18.5)	13 (13.0)	12 (13.5)
Recent trauma^a^	15 (5.3)	7 (7.6)	6 (6.0)	2 (2.2)
Recent cast therapy^a^	5 (1.8)	3 (3.3)	2 (2.0)	0 (0)
Recent travel > 8 hours^a^	4 (1.4)	3 (3.3)	1 (1.0)	0 (0)
Concurrent malignancy	88 (31.3)	24 (26.1)	45 (45.0)	19 (21.3)
Malignancy diagnosed within 2 years of VTE event	7 (2.5)	2 (2.2)	4 (4.0)	1 (1.1)
Central venous line	27 (9.6)	13 (14.1)	12 (12.0)	2 (2.2)
Positive heredity for VTE	7 (2.5)	5 (5.4)	2 (2.0)	0 (0)
Coagulation disorder	7 (2.5)	7 (7.6)	0 (0)	0 (0)
Hormone therapy^b^	7 (4.6)	5 (11.1)	0 (0)	2 (3.2)
Pregnancy^b^	0 (0)	0 (0)	0 (0)	0 (0)
Postpartum^b^	1 (0.7)	1 (2.2)	0 (0)	0 (0)
**No risk factors**	116 (41.3)	29 (31.5)	35 (35.0)	52 (58.4)
**VTE prophylaxis at diagnosis**	11 (3.9)	2 (2.2)	5 (5.0)	4 (4.5)

Values represent the mean ± SD or n (%); VTE = venous thromboembolism.

^a^ = Within 60 days before the VTE event.

^b^ = Percentage of female patients only.

### Risk factors

The prevalence of common risk factors for first-time VTE in different age groups is presented in Table 1. The most common risk factors were recent hospitalization and concurrent malignancy. Surgery, immobilization, and trauma occurred more frequently among those aged 18-64 years compared to the older age groups. Of the patients with first-time VTE, 11 (3.9%) were taking pharmacological VTE prophylaxis at the time of diagnosis. The proportion of first-time VTE cases with none of the registered risk factors increased with age, and it comprised 58% of the oldest age group. The risk factor profile in the 62 patients with recurrent VTE was similar to the risk factor profile in the patients with first-time VTE.

### Thrombosis location

Of the 343 patients with VTE, 161 had pulmonary embolism, 157 had lower extremity DVTs, 10 had upper extremity DVTs and 15 had VTEs at other locations. Of the 281 patients with first-time VTE, 135 had pulmonary embolism, 123 had lower extremity DVTs, 9 had upper extremity DVTs, and 14 had VTEs at other locations (Table 2). First-time iliac DVTs occurred most frequently in the oldest age group, and first-time femoropopliteal DVTs occurred most frequently in the youngest age group. The proportion of first-time pulmonary embolism increased with increasing age. In patients with first-time pulmonary embolism, 55% had bilateral emboli. Unilateral first-time pulmonary embolism occurred predominantly on the right side. First-time VTEs in other locations occurred most frequently in the youngest age group. First-time lower extremity DVTs occurred more frequently on the left-side than on the right-side, in both men and women. A concurrent verified lower extremity DVT was found in 19 patients with first-time pulmonary embolism. Two patients with first-time pulmonary embolism had symptoms and clinical signs of a lower extremity DVT. There were seven cases of first-time abdominal VTE, and seven cases of first-time VTE in the central nervous system.

**Table 2:** Location of thrombosis by age group in patients with first-time VTE

**Disorder and location**	**All (n = 281)**	**18-64 years (n = 92)**	**65-79 years (n = 100)**	**80 years and up (n = 89)**
**Deep vein thrombosis**	**132 (47.0)**	**45 (48.9)**	**47 (47.0)**	**40 (44.9)**
Iliac DVT^a^	52 (39.4)	8 (17.8)	20 (42.6)	24 (60.0)
Femoropopliteal DVT^a^	46 (34.8)	21 (46.7)	14 (29.8)	11 (27.5)
Calf DVT^a^	20 (15.2)	9 (20.0)	8 (17.0)	3 (7.5)
Muscular DVT^a^	5 (3.8)	3 (6.7)	1 (2.1)	1 (2.5)
Upper extremity DVT^a^	9 (6.8)	4 (8.9)	4 (8.5)	1 (2.5)
**Pulmonary embolism**	**135 (48.0)**	**40 (43.5)**	**48 (48.0)**	**47 (52.8)**
Right side PE^b^	36 (26.7)	7 (17.5)	16 (33.3)	13 (27.7)
Left side PE^b^	11 (8.1)	2 (5.0)	4 (8.3)	5 (10.6)
Bilateral PE^b^	74 (54.8)	26 (65.0)	21 (43.8)	27 (57.4)
Unknown location of PE^b^	14 (10.4)	5 (12.5)	7 (14.6)	2 (4.3)
**VTE in another location**	**14 (5.0)**	**7 (7.6)**	**5 (5.0)**	**2 (2.2)**
Abdominal VTE^c^	7 (50.0)	3 (42.9)	4 (80.0)	0 (0)
Central nervous system VTE^c^	7 (50.0)	4 (57.1)	1 (20.0)	2 (100.0)

Values represent n (%) for each group. DVT = deep vein thrombosis, PE = pulmonary embolism, VTE = venous thromboembolism. Bold lettering indicates the type of embolism, and the list below indicates the specific locations for each type of embolism.

^a^ = Number and percentage of patients with DVT.

^b^ = Number and percentage of patients with PE.

^c^ = Number and percentage of patients with VTE in another location.

### Diagnostic method

Of the 161 patients with pulmonary embolism, the diagnosis was verified in 133 with CT scans, in 5 with high probability ventilation/perfusion scans, in 13 by autopsy, and by clinical signs or symptoms of pulmonary embolism in patients with another verified VTE in 10 patients. In 21 (13%) of the patients with pulmonary embolism, the pulmonary embolism was discovered at imaging performed with another objective than evaluation for pulmonary embolism. Of the 157 patients with lower extremity DVTs, the diagnosis was verified in 121 by ultrasonography, in 33 by venography, and in 3 with CT scans. No patient with a lower extremity DVT was diagnosed by autopsy only. Of the seven patients with abdominal VTEs, the diagnosis was verified in five with CT scans and in two by ultrasonography. Of the eight patients with VTEs in the central nervous system, the diagnosis was verified in seven by MRI and in one with a CT scan. Of the ten patients with upper extremity DVTs, the diagnosis was verified in six by ultrasonography, in three by venography, and in one with a CT scan.

### Evaluation of search methods

With our extensive ICD-10 code search, we identified 517 adult individuals. Of these, 313 individuals had a verified VTE event. Therefore, this method of case identification resulted in 39% false positive cases. Other sources (the mortality registry, the anticoagulant registry, the radiology registry, and additional ICD-10 codes, I74.3 and K55.0) revealed an additional 30 individuals with VTE. The total number of individuals with verified VTE was 343. The extensive search method identified 91% of the cases of verified VTE and thus had a false negative rate of 9%. In the original extensive search, 204 individuals did not have a verified VTE. Of these, some had other conditions, including superficial thrombophlebitis, pulmonary hypertension, arterial thromboembolic disease, or edema of the lower extremities. Others were on a medical checkup on a past VTE event. We also performed a limited search, where we only used the ICD-10 codes, I26 and I80.1-9, for case identification. This search resulted in a 36% false positive rate and a 22% false negative rate for identifying patients with pulmonary embolism and/or DVT of the upper or lower extremities.

## Discussion

This study showed that the incidence of VTE in northern Sweden was 167 per 100,000 per year. The incidence increased with age and was higher in women. Pulmonary embolism and lower extremity DVTs were the most common disease manifestations and had similar incidence rates. In our population, the most common risk factors for first-time VTE were recent hospitalization and concurrent malignancy.

We reviewed other studies that presented data on the incidence of first-time VTEs in different age groups and for men and women separately (Table 3). The reported overall incidence varied between 96 and 143 per 100,000 per year [2,3,6-8,16]. Studies that included children and adolescents generally reported a lower overall incidence, because VTE is rare in those age groups [6-8,16]. In the three studies that only included adults, the incidence rates varied between 130 and 143 per 100,000 per year [2,3]. In individuals aged 60 years and older, the incidence was considerably higher in the American and French studies [6-8,16] compared to the other studies. Potential explanations for this discrepancy may be that the different studies used different case identification strategies. We searched the anticoagulant registry, the radiology registry, and the National Patient Registry for additional ICD-10 codes in a subset of our population. This strategy would have yielded an additional 10% of VTE cases, if applied to our whole population. Nevertheless, the incidence rate in our study would have remained lower than those reported in the three studies with the highest incidence rates among individuals aged 60 years and older. On the other hand, we scrutinized the medical records for the presence of previous VTEs, and we included only cases of first-time VTEs in the incidence comparisons. Misclassifications of recurrent VTE cases would have resulted in an overestimation of the incidence. Alternatively, there may be true variations in incidence rates among different studies due to differences in the risk factor patterns among the populations studied; e.g., prevalence of obesity, smoking habits, and hereditary risk factors [17-19]. It is notable that the three Scandinavian studies with comparable populations reported similar incidence rates.

**Table 3:** Comparison of seven studies that reported VTE incidence by age group

	**Present study**	**Næss et al. **[2]	**Braekkan et al. **[3]	**Oger **[6]	**Silverstein et al. **[7]	**Spencer et al. **[8,16]
Patients with first-time DVT and/or PE included	267	740	327	464	444	1567
Period of data collection	2006	1995-2001	1994-2007	1998-1999	1986-1990	1999, 2001, 2003
Geographic location	Sweden	Norway	Norway	France	United States	United States
Age at DVT and/or PE diagnosis, years^a^	70 ± 15	73 (22-102)	N/A	N/A	N/A	64.6
Age groups included	≥18 years	≥20 years	>24 years	All ages	All ages	All ages
Yearly incidence of first-time DVT and/or PE^b^	130	143	140	136	96^c^	114^f^
Incidence of first-time DVT and/or PE by age group^b^ (age range, years)	54 (18-64)	63 (20-64)	54 (<50)	39 (<60)	55 (15-64)^d^	58 (<65)
	270 (65-74)	252 (65-74)	254 (50-69)	354 (60-74)	356 (65-74)^d^	336 (65-74)
	463 (75-84)	529 (75-84)	570 (≥70)	895 (≥75)	628 (75-84)^d^	538 (75-84)
	668 (≥85)	680 (≥85)			818 (≥85)^d^	781 (≥85)
Yearly incidence of first-time DVT and/or PE in men^b^	119	128	151	114	107^e^	103
Yearly incidence of first-time DVT and/or PE in women^b^	142	158	131	149	88^e^	124
Yearly incidence of first-time DVT ^b^	64	93	91	87	49^c^	95^g^
Yearly incidence of first-time PE^b^	66	50	50	46	47^c^	34^h^

VTE = venous thromboembolism, DVT = deep vein thrombosis, PE = pulmonary embolism, N/A = data not available.

^a^ = Mean age with standard deviation or median age with age range, according to each study.

^b^ = Incidence per 100,000 person years.

^c^ = Incidence age- and sex adjusted.

^d^ = Incidence for each age group calculated as follows: The total number of female residents in the age group was calculated as: x = number of cases in females/the incidence in females. The total number of male residents in the age group was calculated as: y = number of cases in males/the incidence in males. Then, the incidence = the total number of cases in the age group/(x + y).

^e^ = Incidence age adjusted.

^f^ = Age-adjusted VTE incidence rate, all events included.

^g^ = Age-adjusted DVT incidence rate with or without concurrent PE.

^h^ = Age-adjusted PE incidence rate with or without concurrent DVT.

In the reviewed studies, the incidence was (per 100,000 per year) 103 to 151 in men, and 88 to 158 in women (Table 3) [2,3,6-8,16]. Although the overall incidence of VTE in our study was higher in women than in men, the incidence of VTE was higher in men than in women in the 60 to 74 year-old age group. A similar finding was reported in a Norwegian study [2]. Interestingly, we did not detect any differences in the risk factor patterns between men and women in this age group that might explain the difference in incidence. A possible explanation for this finding could be that, compared to men, women might acquire risk factors at a higher age, and this may have contributed to a later VTE incidence peak.

In the present study, half of the first-time VTE events were classified as a pulmonary embolism. This proportion contrasted with findings in most other reviewed studies, which reported a considerably lower incidence of pulmonary embolism compared to DVT [2,3,6,8,16]. The high incidence of pulmonary embolism in our study, compared to other studies, might be attributable to the different study periods. Over the last decade, the use and quality of CT scans has improved for disease diagnosing and monitoring (e.g., in malignant diseases). Another factor that could have contributed to a higher incidence of pulmonary embolism in our study was our inclusion of cases of objectively verified, asymptomatic pulmonary embolism. In one other study, asymptomatic cases were excluded [3]. In addition, the classifications for cases with verified DVT and concomitant symptoms of pulmonary embolism vary between studies. We chose to classify these cases as pulmonary embolism; however, in many studies, these cases were classified as DVT [2,3,7,8]. This can also contribute to a higher incidence of pulmonary embolism in our study. Finally, the autopsy rate can affect the incidence of pulmonary embolism. In the study by Silverstein et al., performed between 1986 and 1990, the autopsy rate was 30-35%; this high rate could have contributed to the high incidence of pulmonary embolism reported in their study [7]. It is known that the autopsy rate has decreased over time [7]. In the County of Västerbotten, the autopsy rate was 17% in 2006, and only 13 of the 161 patients with pulmonary embolism were diagnosed at autopsy.

Recent hospitalization was the most common risk factor in all age groups, and the proportion of recently hospitalized patients with VTE was similar across all age groups. In our study, 31% of patients with first-time VTE had a concurrent malignancy; this proportion was highest in the 65-79 year-old age group. In comparison, in the large Worchester VTE study, the proportion of patients with concurrent malignancy was 30% [8]. Of the total population of patients with first-time VTE, we could not identify any risk factor in 41%. This proportion is lower than in other Scandinavian studies [2,3,5], but higher than in a large American study [8].

The primary strength of our study was the use of an extensive search method with a wide range of ICD-10 diagnostic codes. This approach enabled the identification of as many VTE cases as possible. In addition, all identified VTE cases were verified. We also evaluated a limited ICD-10 code search method with aim of identifying patients with lower or upper extremity DVTs or pulmonary embolism; again, we verified all cases. The proportion of false positive cases found with this search method was 36%. In a Danish study that used a similar limited search method, the proportion of false positive cases was 42% [20]. This high proportion of false positive cases emphasizes the importance of case verification when using diagnostic codes to identify patients with VTE.

Our study was performed in a geographically well-defined area; we included both inpatient and outpatient cases of VTE and had no upper age limit for inclusion. One weakness of the study was the retrospective study method. We only had access to data available from medical records. Thus, the information about risk factors and previous VTE events was limited to the content of the medical records. Due to incomplete information on risk factors in medical records, it is possible that we have underestimated the prevalence of risk factors at time of VTE diagnosis. Another limitation was that our results were based on a limited number of VTE events, which restricted the power to detect small differences between groups.

## Conclusion

The incidence of VTE was 167 per 100,000 person years, and it increased with age. The incidence was highest among older women. Malignancy and hospitalization were the most prevalent risk factors for VTE. We found a high incidence of pulmonary embolism compared to other studies.

## Abbreviations

VTE: Venous thromboembolism; DVT: Deep vein thrombosis; ICD: International Classification of Diseases; CT: Computed tomography; MRI: Magnetic resonance imaging.

## Competing interests

The authors declare that they have no competing interests.

## Authors’ contributions

LJ and ML designed the study. All authors added important intellectual content, participated in the analysis and interpretation of data, participated in drafting the manuscript and approved the final version of the manuscript.
